# Supplementary material for: BARCODE: high throughput screening and analysis of soft active materials
Source: Nat Commun. 2025 Dec 31;17:1192. doi: 10.1038/s41467-025-67963-3 (PMC12858825; doi:10.1038/s41467-025-67963-3)
Supplement: Supplementary file 1 — Supplementary Information [file 41467_2025_67963_MOESM1_ESM.pdf]

BARCODE: high throughput screening and analysis of soft active materials

Qiaopeng Chen[†1], Aditya Sriram[†2], Ayan Das[1], Katarina Matic[2], Maya Hendija[2], Keegan Tonry[3], Jennifer L. Ross[4], Moumita Das[3], Ryan J. McGorty[2], Rae M. Robertson-Anderson[2*], Megan T. Valentine[1*]

[1] Department of Mechanical Engineering, University of California, Santa Barbara
[2] Department of Physics and Biophysics, University of San Diego
[3] School of Physics and Astronomy, Rochester Institute of Technology
[4] Department of Physics, Syracuse University

[†] Equal contribution

[*]Corresponding authors, equal contribution:
randerson@sandiego.edu, valentine@engineering.ucsb.edu.

Supplemental Information

Section S1. Detailed description and formulas for BARCODE parameters

Table S1. BARCODE run time and efficiency metrics for the four different datasets examined in the main text.

Table S2. Statistical analysis of agreement between filaments speeds computed with BARCODE and those reported in doi:10.1093/pnasnexus/pgad245.

Section S1. Description of BARCODE Parameters

The BARCODE platform is divided into 3 independent branches: the image binarization (IB) branch, intensity distribution (ID) branch, and optical flow (OF) branch. The 17 specific parameters computed across all branches, described below, are displayed in a color-scaled array (see Fig. 2E reproduced below) to allow for rapid inspection and categorization. The numerical values that encode the colorized array, as well as information-rich reduced data structures (RDS) from which the parameters are determined, are also saved for further quantitative physics-driven and data-driven analyses.

Image Binarization (IB) Branch: Connectivity, Maximum Island Area, Maximum Void Area, Maximum Island Area Change, Maximum Void Area Change, Initial Maximum Island Area, Initial 2nd Maximum Island Area

Intensity Distribution (ID) Branch: Maximum Kurtosis, Maximum Median Skewness, Maximum Mode Skewness, Kurtosis Change, Median Skewness Change, Mode Skewness Change

Optical Flow (OF) Branch: Speed, Speed Change, Flow Direction, Directional Spread

BARCODE assumes that the data inputs are stacks of 2D images (frames) acquired over time (i.e., videos, tiff files) using optical microscopy. Input video files can have multiple channels, corresponding to specific components in the materials, which are analyzed as separate videos. Each frame of a video can be described as a $m \times n$ matrix of pixels that reports the intensity value at each pixel position $B(x, y)$ where $0 \leq x \leq m$ and $0 \leq y \leq n$. Each frame i^{th} is then expressed as a matrix of $B(x, y, i)$ values. To increase processing speed and reduce the data size, it is possible to downsample in time, by choosing to analyze every k^{th} frame. Additionally, in the OF branch, it is possible to downsample in space, by averaging together $p \times p$ windows of pixels to result in a stack of T/k images of $m/p \times n/p$ pixels. By default, $k = 10$ and $p = 8$.

Warnings and Errors

BARCODE may sometimes have difficulty with images that are considered low contrast or which show saturation. To define low contrast images, for initial frame $i = 1$ and final frame $i = N_f$, the mean pixel intensity $\overline{B(i)}$ and minimum $B_{min}(i)$ is calculated. Low contrast (or ”dim”) video files are defined such that $\frac{2}{e} \cdot \overline{B(i)} \leq B_{min}(i)$ for both the initial and final frame. Users can choose to analyze images classified as dim, which produces a flag in the CSV data output indicating the video has been labeled as dim, represented as a Flag $= 1$ value for a given video. Otherwise, videos with this classification are skipped due to potential unreliability of the outputs.

BARCODE can also detect saturation in a video, defined here as the case where the maximum pixel intensity $B_{max}(i)$ is equal to the mode pixel intensity value B_i^{mode} for each frame i. This produces

a flag in the CSV data output indicating the video has been labeled as saturated, represented as a Flag $= 2$ value.

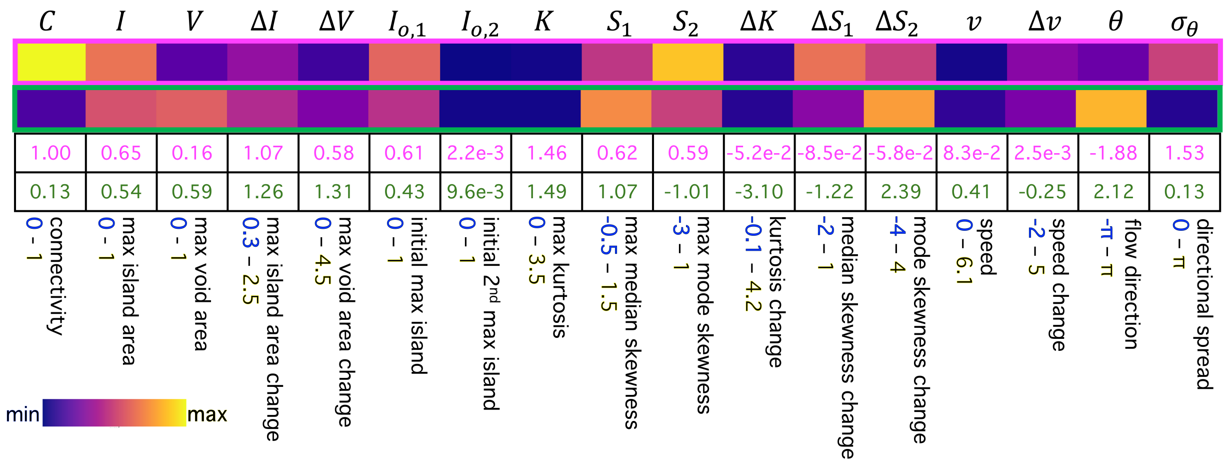

Figure 1: **Sample BARCODE outputs with names and variables of parameters.** Two example barcodes, reproduced from Figure 2E, are shown.

1 Image Binarization Branch

We binarize each frame of the video using a threshold value of $\overline{B(i)} \times (1 + \text{offset})$ where the offset is user-inputted with a default value of 0.1 and $\overline{B(i)}$ is the mean intensity of the ith original image, averaged across all pixel positions:

$$\overline{B(i)} = \frac{1}{N} \sum_{x,y} B(x, y, i) \tag{1}$$

Here, N is the total number of pixels in the image of size $m \times n$. The intensity of each pixel in the ith frame of the binarized image is given by:

$$b(x, y, i) = \begin{cases} 1, & \text{if } B(x, y, i) \geq \overline{B(i)} \times (1 + \text{offset}) \\ 0, & \text{if } B(x, y, i) < \overline{B(i)} \times (1 + \text{offset}) \end{cases} \tag{2}$$

Each parameter computed in this branch, described below, is derived from analysis of the stack of binarized images, which are saved as RDS.

1.1 Connectivity C

Connectivity C evaluates the persistence of percolated pathways across a material by determining whether a continuous path of $b = 1$ pixels exists across the x or y axis in each frame. A frame with

or without a percolated path is assigned a connectivity of $C_i = 1$ or 0. We determine the number of frames with $C_i = 1$ and divide by the total number of frames N_f to determine a fractional measure of connectivity C for each video, which we report in the barcode. To compute C_i, we label the connected $b = 1$ areas in the binarized images using scipy.ndimage.label, a labeling function provided by the open source python image processing tool scipy [1]:

$$L(x, y, i) = \text{label}(b(x, y, i)) \tag{3}$$

As a result, each distinct connected region in the binary image $b(x, y, i)$ is assigned a unique label number, as determined by analysis of connectivity of each pixel with its nearest neighbors. We then record the labels of the connected areas on the boundaries of the image of size $m \times n$:

$$\begin{aligned} L_{y=0} &= \{L(x, y) \mid y = 0\} \\ L_{y=n-1} &= \{L(x, y) \mid y = n-1\} \\ L_{x=0} &= \{L(x, y) \mid x = 0\} \\ L_{x=m-1} &= \{L(x, y) \mid x = m-1\} \end{aligned}$$

Next, we determine the connectivity by determining whether a single label persists from one external boundary to the other in the x or y direction. In practice, this is accomplished by calculating the intersection of $L_{y=n-1}$ and $L_{y=0}$ as well as the intersection of $L_{x=m-1}$ and $L_{x=0}$ for each frame of the binarized video. $C_i = 1$ if there is a single cluster (identified by a single label) that spans the entire image in either x or y directions, and $C_i = 0$ if no such cluster exists, mathematically expressed as

$$C_i = \begin{cases} 1, & \text{if } L_{y=n-1} \cap L_{y=0} \neq \emptyset \text{ or } L_{x=0} \cap L_{x=m-1} \neq \emptyset \\ 0, & \text{otherwise} \end{cases} \tag{4}$$

To determine the connectivity C for a given video, we compute the average C_i value across all frames:

$$C = \frac{1}{N_f} \sum_i C_i \tag{5}$$

1.2 Maximum island area I

The maximum island area I is defined as the area of the largest contiguous region of white ($b = 1$ pixels) over the entire binarized video (see Eq. 2), which we report as the fraction of the area of the FOV. To compute I, we calculate the area of each distinct connected $b = 1$ region in the labeled image $L(x, y, i)$ (see Eq. 3). Let I_i^k represent the area (in pixels) of the kth connected region in frame i, where the k index is sorted such that $k = 1$ represents the largest connected white region, $k = 2$ represents the second largest white region, etc. In each case, we normalize by the total number of pixels N. We determine I by evaluating the maximum I_i^1 value across all frames. To produce a more statistically robust measure of I, we compute and report the mean of the highest 10% of I_i^1 values in the videos:

$$I = \frac{1}{0.1 N_f} \sum_{I_i^1 \in \text{highest } 10\%} \frac{I_i^1}{N} \tag{6}$$

1.3 Maximum void area V

The maximum void area V is the complement to the maximum island area that identifies and computes the areas of the largest contiguous regions of $b = 0$ (black) pixels in each frame of the binarized video. The computation is identical to that described above but performed on $b = 0$ rather than $b = 1$ pixels.

1.4 Maximum island area change ΔI

The maximum island area change ΔI quantifies the relative change in the area of the maximum island at the end compared to the beginning of the video. We define this quantity as the ratio of the average maximum island area over a user-defined fraction of final frames to that of the average maximum island area over the user-defined fraction of initial of frames. X is a user-defined parameter to specify the fraction, which by default is set to $X = 0.05$ in Equation (7). This parameter can therefore be expressed as:

$$\Delta I = \frac{\langle I_i^1([(1-X)N_f] : N_f)\rangle}{\langle I_i^1(0 : XN_f)\rangle} \tag{7}$$

where the average maximum island area over a given range of frames i_a to i_b is computed as

$$\langle I_i^1(i_a : i_b)\rangle = \frac{1}{b-a}\sum_{i_b}^{i_a} I_i^1 \tag{8}$$

1.5 Maximum void area change ΔV

The maximum void area change ΔV is the complement to the island area change that quantifies the relative change in the area of the maximum void at the end compared to the beginning of the video. The computation is identical to that described above but performed on $b = 0$ rather than $b = 1$ pixels.

1.6 Initial maximum and initial 2nd maximum island

The initial maximum island, $I_{0,1}$, is defined as the average of the largest island areas I_i^1 in a user-defined fraction of initial frames in the video. The initial 2nd maximum island, $I_{0,2}$, is defined as the average of the 2nd largest island areas I_i^2 in a user-defined fraction of initial frames in the video. X is a user-defined parameter to define this fraction, which by default is set to $X = 0.05$.

To calculate these parameters, we sort all connected $b = 1$ regions in frame i, i.e., I_i^1, in order of descending area. Then, the initial island area and initial 2nd island area are given by:

$$I_{0,1} = \frac{1}{XN_f}\sum_{i=1}^{XN_f} I_i^1 \tag{9}$$

$$I_{0,1} = \frac{1}{XN_f} \sum_{i=1}^{XN_f} I_i^2 \tag{10}$$

$$I_{0,1} = \frac{1}{XN_f} \sum_{i \in \text{ first } 100X\%} I_i^1 \tag{11}$$

$$I_{0,2} = \frac{1}{XN_f} \sum_{i \in \text{ first } 100X\%} I_i^2 \tag{12}$$

2 Intensity Distribution Branch

In this module, we analyze the properties of the distribution of pixel intensities within a frame, as well as how this distribution changes over time.

The intensity distribution curve $f(B)$ is a continuous function representing the probability of occurrence of intensity B, and is calculated for the i_{th} frame as follows:

$$f(B) = \frac{1}{N} \sum_{x,y} \delta(B(x,y) - B) \tag{13}$$

where: N is the total number of pixels in the image of size $m \times n$, and $\delta(B(x,y) - B)$ is the Dirac delta function, which is 1 if $B(x,y) = B$, and 0 otherwise. In practice, commonly the Dirac delta function is replaced with a bin width of finite size to produce a discrete histogram of values.

To represent the intensity distribution curve, we calculate from a user-defined number of bins n_b the edges of the bin. For each bin n_j, we then count the number of pixels $C(B_{n_j})$ for all intensity values B within the bin. For each bin, we divide by the total number of pixel counts to produce a normalized probability distribution $P(B_{n_j}) = \frac{C(B_{n_j})}{\Sigma_{n_j} C(B_{n_j})}$. We remove all bins with probability less than a user-defined noise threshold, which is set to 0.0005 by default, and renormalize to ensure $\Sigma_{n_j} P(B_{n_j}) = 1$.

We then calculate the moments of the probability distribution for a given frame i as follows:

$$\text{Mean: } \mu_i = \mathbb{E}[B] = \Sigma_{n_j} B_{n_j} P(B_{n_j})$$

$$\text{Centralized Moment: } \mu_{i,m} = \mathbb{E}[(B - \mu_i)^m] = \Sigma_{n_j} (B_{n_j} - \mu)^m P(B_{n_j})$$

$$\text{Variance: } \sigma_i^2 = \mu_{i,2}$$

We also calculate the median B_i^{median} as the first value of n_j at which the cumulative probability exceeds 0.5 and the mode B_i^{mode} as the value at which the probability is maximal.

2.1 Maximum kurtosis K

To analyze the shape of the intensity distribution, we calculate kurtosis. A high value of kurtosis indicates that the distribution contains heavier tails compared to a normal Gaussian curve. We calculate the kurtosis for a given frame i as $K_i = \frac{\mu_{i,4}}{\sigma_i^4} - 3$.
The motivation for calculating maximum kurtosis stems from the need to capture extreme behavior within the intensity distribution during network remodeling. Maximum kurtosis is defined as the average of the highest 10% kurtosis values in within the video sequence.

$$K = \frac{1}{0.1N_f} \sum_{K(i) \in \text{ top } 10\%} K(i) \tag{14}$$

2.2 Maximum median skewness S_1

To quantify asymmetry, we compute the median skewness. Skewness is the parameter that describes the shape of one curve, by measuring the asymmetry of the intensity distribution curve relative to the mean. A high positive (or negative) skewness value indicates that the distribution has a long tail of high (or low) intensities relative to the mean, whereas a skewness value near zero indicates that the intensity distribution is more symmetric around its mean. The median skewness for the ith frame is defined as:

$$S_1(i) = 3 \times \frac{\mu_i - B_i^{\text{median}}}{\sigma_i} \tag{15}$$

The maximum median skewness, denoted S_1, is calculated by taking the average of the highest 10% median skewness values:

$$S_1 = \frac{1}{0.1N_f} \sum_{S_1(i) \in \text{ top } 10\%} S_1(i) \tag{16}$$

2.3 Maximum mode skewness S_2

The mode skewness is defined as:

$$S_2(i) = \frac{\mu_i - B_i^{mode}}{\sigma_i} \tag{17}$$

To compute the maximum mode skewness, we calculate the average of the highest 10% mode skewness values:

$$S_2 = \frac{1}{0.1N_f} \sum_{S_2(i) \in \text{top } 10\%} S_2(i) \tag{18}$$

2.4 Kurtosis change ΔK

To quantify how the material evolves over time, we analyze the change in kurtosis between a user-defined fraction of the final and initial frames of the video sequence, which enables us to identify any changes in the distribution's extrema as the network progresses over time:

$$\Delta K = \langle K((1-X)N_f : N_f)\rangle - \langle K(0 : XN_f)\rangle \tag{19}$$

where $\langle K((1-X)N_f : N_f)\rangle$ and $\langle K(0 : XN_f)\rangle$ are defined similar to Eq. (8). X is the user-defined fraction which is set to $X = 0.05$ by default.

2.5 Median skewness change ΔS_1

To quantify how the structure evolves over time, we analyze the change in median skewness between the final and initial frames of the video sequence to capture any shifts in the distribution asymmetry.

$$\Delta S_1 = \langle S_1((1-X)N_f : N_f)\rangle - \langle S_1(0 : XN_f)\rangle \tag{20}$$

where $\langle S_1((1-X)N_f : N_f)\rangle$ and $\langle S_1(0 : XN_f)\rangle$ are calculated for the final and initial fraction of frames, respectively, defined similarly to Eq. (8) and using a user-defined fraction X, which is set to $X = 0.05$ by default.

2.6 Mode skewness change ΔS_2

We also analyze the change in mode skewness between the final and initial frames of the video sequence to capture any shifts in the distribution asymmetry. X is the user-defined fraction of frames, which is set to $X = 0.05$ by default.

$$\Delta S_2 = \langle S_2((1-X)N_f : N_f)\rangle - \langle S_2(0 : XN_f)\rangle \tag{21}$$

3 Optical Flow Branch

To measure the dynamics of active materials, we implemented the Farneback optical flow algorithm, using the function "cv.calcOpticalFlowFarneback" from OpenCV [3]. The output is a dense optical flow field represented as a 2-dimensional array. For each frame pair in a video, the format of output flow is an array of shape (m/p,n/p,2):

$$flow[x, y] = \begin{bmatrix} u_{xy} \\ v_{xy} \end{bmatrix} \tag{22}$$

where u_{xy} and v_{xy} represent the horizontal and vertical displacement respectively, given in units of pixels.

3.1 Speed v

To analyze the flow properties of a material sample across an entire video, we computed average metrics by averaging the flow fields over multiple frame pairs $(i, i + \Delta i)$, where Δi is the effective time step (given by a discrete number of frames) between frame pairs; Δi is a parameter provided by the user. For a given frame pair $(i, i + \Delta i)$, the flow field is calculated as:

$$flow_i[x, y] = \begin{bmatrix} u^i_{xy} \\ v^i_{xy} \end{bmatrix} \tag{23}$$

Then the speed v is calculated as the average magnitude of the displacement vectors, averaged over all vectors of all flow fields:

$$V^i_{xy} = \frac{\sqrt{(u^i_{xy})^2 + (v^i_{xy})^2}}{\Delta i} \tag{24}$$

$$v(i) = \frac{1}{N_v} \sum_{x=1}^{m/p} \sum_{y=1}^{n/p} V^i_{xy} \tag{25}$$

$$v = \frac{1}{N_f} \sum_{i=1}^{N_f} v(i) \tag{26}$$

where $N_v = \frac{m \times n}{p^2}$ is the total number of vectors in each frame, and i represents the ith frame in the video. The speeds, initially calculated in units of pixels/frame, are converted to units of distance/time using user-inputted values of the pixel-to-micron conversion and frame rate.

3.2 Speed change Δv

To capture the extent to which the dynamics change over the course of the video, we calculate the speed change Δv, defined as the change in the speed between the last $X\%$ of frames and the first $X\%$ of frames:

$$\Delta v = \langle v((1 - X)N_f : N_f)\rangle - \langle v(0 : XN_f)\rangle \tag{27}$$

where $\langle v((1 - X)N_f : N_f)\rangle$ and $\langle v(0 : XN_f)\rangle$ are defined similar to Eq. (8). X is the user-defined fraction which is set to $X = 0.05$ by default.

3.3 Flow direction θ

The average direction θ is calculated in two steps: First, for a certain frame i, given a set of angles $\{\theta^i_{xy}\}$ at every pixel, each angle is first represented as a unit vector on the unit circle:

$$\tilde{\mathbf{V}}^i_{xy} = (\cos\theta^i_{xy},\ \sin\theta^i_{xy}) = (v^i_{xy}, u^i_{xy})/\sqrt{(v^i_{xy})^2 + (u^i_{xy})^2}. \tag{28}$$

The average of these unit vectors captured in both the x and y directions is calculated per frame, and averaged across each frame. Then, the angle of flow direction was calculated using the two-argument arctan2 function, which correctly determines the angle in all four quadrants by using the signs of both x and y.

$$\theta = arctan2(\frac{1}{N_v N_f}\sum_{i=1}^{N_f}\sum_{x=1}^{m/p}\sum_{y=1}^{n/p}\sin\theta^i_{xy}, \frac{1}{N_v N_f}\sum_{i=1}^{N_f}\sum_{x=1}^{m/p}\sum_{y=1}^{n/p}\cos\theta^i_{xy}) \tag{29}$$

The result is thus returned in the full range of $-\pi < \theta \leq \pi$. This approach of taking the average of the unit velocity vector components $\cos\theta^i_{xy}$ and $\sin\theta^i_{xy}$ before determining the flow direction θ, prevents complications due to discontinuities along the periodic boundary.

3.4 Directional spread

The mean resultant length of the unit velocity vector is given by

$$L^i = \|\frac{1}{N_v}\sum_{x=1}^{m/p}\sum_{y=1}^{n/p}\tilde{\mathbf{V}}^i_{xy}\|. \tag{30}$$

The value of L^i reports the angular distribution: $L^i \to 1$ when all vectors point in the same direction (perfect alignment), and $L^i \to 0$ when the vectors are uniformly distributed (completely random). We define the directional spread σ_θ as using the circular standard deviation, which can be computed from L^i and averaged over all frames as follows

$$\sigma_\theta = \frac{1}{N_f}\sum_{i=1}^{N_f}\sqrt{-2\ln L^i}, \tag{31}$$

which gives $\sigma_\theta = 0$ for perfectly aligned vectors ($L^i = 1$), while σ_θ increases for vectors showing deviations from perfect alignment. In the limit of perfect uniformity, σ_θ diverges as $L^i \to 0$. In practice, we find most values of $\sigma_\theta < \pi$.

Table S1. BARCODE run time and efficiency metrics for the four different datasets examined in the main text.

Dataset	active cytoskeleton composite	actomyosin network	kinesin-driven composite	hdF cell monolayer	MCF10A cell monolayer
**source**	*	**	see methods	***	see methods
**barcode**	Fig 3C,D	Fig 4B,C	Fig 4I	Fig 5B	Fig 5H
**# of videos**	48	132	131	72	20
**# of channels**	2	2	1	2	2
**avg. frames per video**	979	394	265	117	829
**frame size (pixels)**	256×256	1024×1024	256×256	960×608	512×512
**avg. video size (MB)**	123	775	364	131	207
**dataset size (GB)**	11.8	200	44.6	18.4	8.1
**barcode run time (mins)**	10.5	31	75.6	74.2	11.6
**avg. run time per video (s)**	6.6	19.4	41.6	30.9	17.4
**avg. run time per GB (s)**	50	47	170	240	80

*doi:10.1093/pnasnexus/pgad245
**doi:10.1038/nphys2715
***doi:10.1098/rsif.2023.0160

Table S2. Statistical analysis of agreement between filaments speeds computed with BARCODE and those reported in doi:10.1093/pnasnexus/pgad245. Analysis of the Pearson correlation coefficient shows a high degree of statistical agreement between the two datasets for all channels (actin, microtubule) and dynamical classes (slow, fast, multimode). Statistical analysis was performed in Python using a two-sided Pearson correlation test with the software package scipy.stats. Each sample within each data set represents an independent field of view. The sample sizes were as follows: n = 12 (slow), n =30 (fast), n = 6 (multimode).

data class	all	slow	fast	multimode
**both channels**				
Pearson coefficient	0.99	0.87	0.99	0.99
p-value	4.5×10^{-85}	2.7×10^{-8}	3.9×10^{-54}	1.0×10^{-10}
**actin channel**				
Pearson coefficient	0.98	0.89	0.98	0.99
p-value	1.1×10^{-40}	1.0×10^{-4}	5.0×10^{-25}	3.9×10^{-6}
**microtubule channel**				
Pearson coefficient	0.99	0.86	0.99	0.99
p-value	1.0×10^{-44}	3.7×10^{-4}	9.6×10^{-30}	1.6×10^{-4}

References

[1] Scipy. NDImage Documentation. [Online]. https://docs.scipy.org/doc/scipy/reference/ndimage.html. (2025)

[2] Zwillinger, D. and Kokoska, S. (2000). CRC Standard Probability and Statistics Tables and Formulae. Chapman & Hall: New York. 2000. https://docs.scipy.org/doc/scipy/reference/generated/scipy.stats.kurtosis.html (2025)

[3] OpenCV. calcOpticalFlowFarneback Documentation. [Online]. https://docs.opencv.org/3.4/dc/d6b/group__video__track.html. (2024)
